# *In Vitro* Secondary Structure of the Genomic RNA of Satellite Tobacco Mosaic Virus

**DOI:** 10.1371/journal.pone.0054384

**Published:** 2013-01-22

**Authors:** Shreyas S. Athavale, J. Jared Gossett, Jessica C. Bowman, Nicholas V. Hud, Loren Dean Williams, Stephen C. Harvey

**Affiliations:** 1 School of Biology, Georgia Institute of Technology, Atlanta, Georgia, United States of America; 2 School of Chemistry and Biochemistry, Georgia Institute of Technology, Atlanta, Georgia, United States of America; 3 School of Computational Science and Engineering, Georgia Institute of Technology, Atlanta, Georgia, United States of America; University of Kansas Medical Center, United States of America

## Abstract

Satellite tobacco mosaic virus (STMV) is a *T* = 1 icosahedral virus with a single-stranded RNA genome. It is widely accepted that the RNA genome plays an important structural role during assembly of the STMV virion. While the encapsidated form of the RNA has been extensively studied, less is known about the structure of the free RNA, aside from a purported tRNA-like structure at the 3′ end. Here we use selective 2′-hydroxyl acylation analyzed by primer extension (SHAPE) analysis to examine the secondary structure of *in vitro* transcribed STMV RNA. The predicted secondary structure is unusual in the sense that it is highly extended, which could be significant for protecting the RNA from degradation. The SHAPE data are also consistent with the previously predicted tRNA-like fold at the 3′ end of the molecule, which is also known to hinder degradation. Our data are not consistent with the secondary structure proposed for the encapsidated RNA by Schroeder *et al.*, suggesting that, if the Schroeder structure is correct, either the RNA is packaged as it emerges from the replication complex, or the RNA undergoes extensive refolding upon encapsidation. We also consider the alternative, *i.e.*, that the structure of the encapsidated STMV RNA might be the same as the *in vitro* structure presented here, and we examine how this structure might be organized in the virus. This possibility is not rigorously ruled out by the available data, so it remains open to examination by experiment.

## Introduction

Satellite tobacco mosaic virus (STMV) is a *T* = 1 icosahedral virus with a single-stranded, positive-sense RNA genome, 1058 nucleotides in length. A capsid of 60 identical protein subunits surrounds the genome in the STMV particle. Like other satellite viruses, STMV encodes its own capsid protein but requires a helper virus for replication. For a review on the general properties of STMV, see Dodds [1]. STMV has been studied extensively as a model for the assembly of other single-stranded RNA viruses [2], and as a vector for the delivery of foreign genes into tobacco plants [3].

**Figure 1:**
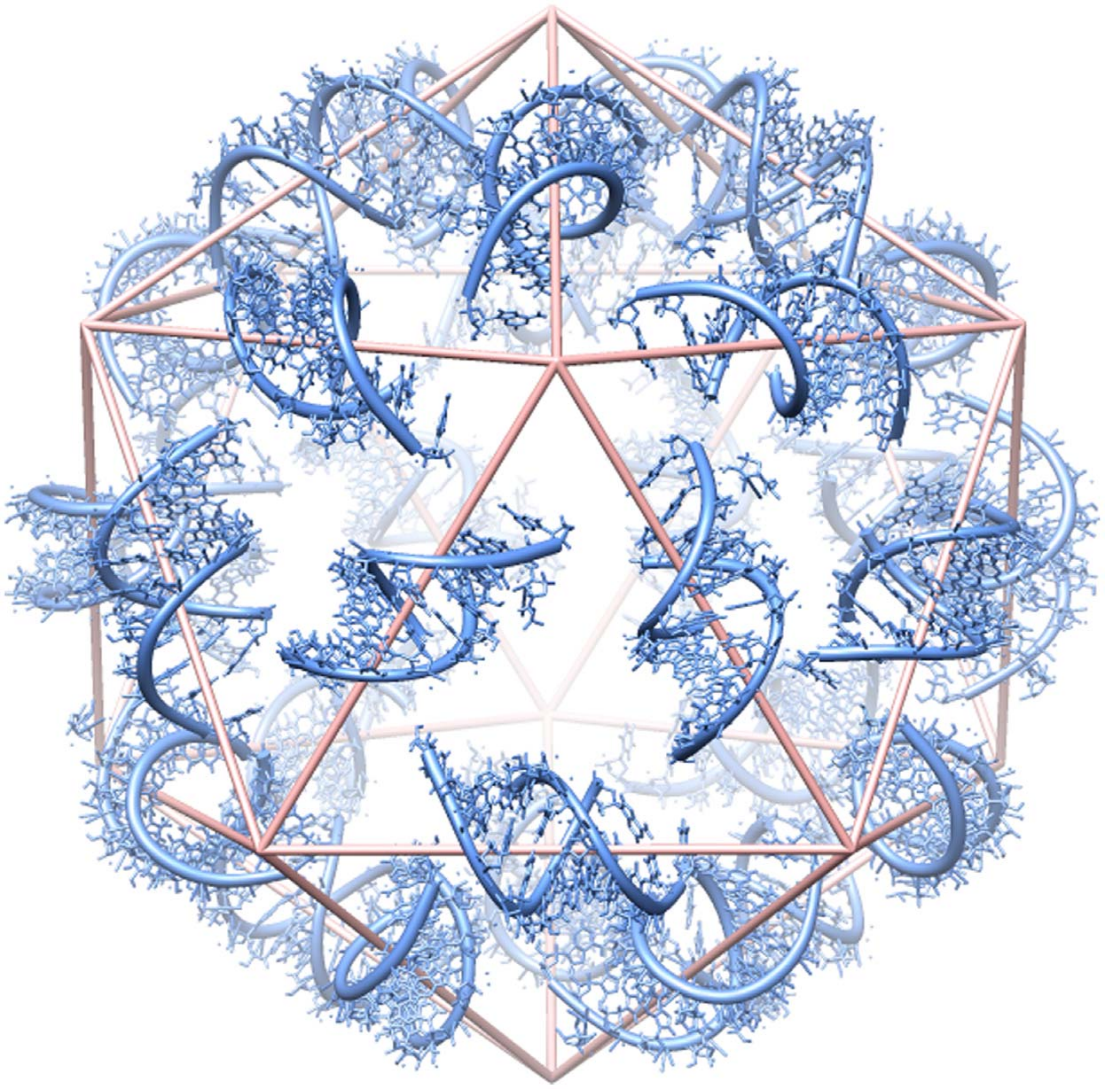
Distribution of double-helical RNA segments in the STMV virion. The crystal structure of STMV [4] reveals 30 segments of double-helical RNA (blue). Each helix contains 9 base pairs, centered on a crystallographic two-fold axis. An icosahedral cage (pink) is shown for reference. Adopted from [8].

Efforts to characterize the RNA and its role in assembly have produced mixed results. The virus crystal structure has been solved at 1.8 Å resolution [4], although some of the protein and 41% of the RNA are not visible in the electron density maps. The RNA that is visible is revealed as 30 double-helical segments, each 9 base pairs in length and closely associated with dimers of coat protein (Figure 1). The helical axes are perpendicular to the icosahedral 2-fold axes, forming part of the edges of an icosahedron. With this constraint on the structure, Larson and McPherson proposed that the RNA forms a series of stem-loop substructures, with only short-range (local) base pairing. They suggested that coat proteins bind to successive stem-loops as these form upon emerging from the replication complex [5]. The results of atomic force microscopy (AFM) experiments are consistent with this hypothesis [6].

Schroeder *et al.* used chemical probing to examine the RNA structure inside the virus. They combined these data with the assumption of co-replicational folding to produce an ensemble of models for the secondary structure [7]. Each of these contains a series of 30 stem loops, with local base pairing; it is important to emphasize that the absence of long-range base pairs is an assumption built into the model, not a hypothesis that was tested by the chemical probing. They reported a single “most representative” secondary structure from that ensemble. We recently used that secondary structure to develop an all-atom model for the mature virus [8], containing every single amino acid and every single nucleotide. (We believe this is the first such model for any virus.).

The capsid-free form of STMV RNA has been relatively overlooked in structural studies, in part because the secondary structure of the encapsidated RNA is believed to be different than the free RNA [5]. A tRNA-like structure (TLS) has been predicted at the 3′ end of the molecule [9], [10], but there is no evidence in the crystallographic data for or against its existence in the encapsidated RNA. A feature seen in AFM images of phenol extracted RNA could be interpreted as the predicted TLS [6], but Schroeder *et al.* have concluded that the TLS is not compatible with their chemical modification data [7]. Larson *et al.* have argued that, if the tRNA-like structure and replication recognition site structure were maintained inside the virus, there would be insufficient RNA remaining to connect the stem-loop segments [4].

Here we report a secondary structure model for *in vitro* transcribed STMV RNA, based on chemical probing data obtained using selective 2′-hydroxyl acylation analyzed by primer extension (SHAPE) [11]. SHAPE provides information on local nucleotide dynamics [12], thus reflecting the extent to which a nucleotide is constrained by base pairing or other interactions [13]. The SHAPE signal is highly correlated with Watson-Crick base pairing [14], and is capable of significantly improving the accuracy of RNA secondary structure predictions [13]. Our primary motivation for this work is to establish the secondary structure for the free STMV RNA, in the absence of the capsid protein. We also compare our probing data to the secondary structure proposed by Schroeder *et al.* for the RNA *in virio*, [7], and to the predicted tRNA-like structure at the 3′ end of the RNA [9], [10].

## Results and Discussion

### SHAPE Analysis of the Free form of STMV RNA

SHAPE [11] involves treating the RNA with an electrophilic reagent that reacts selectively at the ribose 2′-OH position of conformationally flexible nucleotides to form 2′-*O*-adducts. Reverse transcription using fluorescently labeled primers gives cDNA fragments whose lengths are determined by locations of the 2′-*O*-adducts, and whose quantities can be measured by capillary electrophoresis.

We first probed the *in vitro* transcribed STMV RNA in the presence of 250 mM Na^+^ using the SHAPE reagent N-methylisatoic anhydride (NMIA). Under these conditions (no Mg^++^), one would expect the formation of secondary structure, but not necessarily tertiary structure [15]–[17]. We obtained good quality SHAPE reactivity data on 1029 nucleotides, or 97.3% of the 1058-base long STMV RNA. Nucleotides 1–4 and 1034–1058 were omitted from the analysis. The normalized SHAPE reactivity values for STMV RNA ranged from −0.17 to 2.34 with the exception of nucleotide 427, whose reactivity was an outlier at 7.25. Nucleotides with normalized reactivity values <0.3 are considered unreactive; 0.3 to 0.7, moderately reactive; >0.7, highly reactive [13]. Using these criteria, we observed 727 unreactive nucleotides, 189 moderately reactive nucleotides, and 113 highly reactive nucleotides. Six nucleotides –244, 427, 449, 469, 887, and 974– also met the criterion for hyper-reactivity, i.e., normalized reactivity >2 [12]. The data processing procedures are given in more detail in the Methods section, and in the Supporting Information.

### The SHAPE-restrained STMV RNA Secondary Structure Contains Long-range Base Pairing

**Figure 2:**
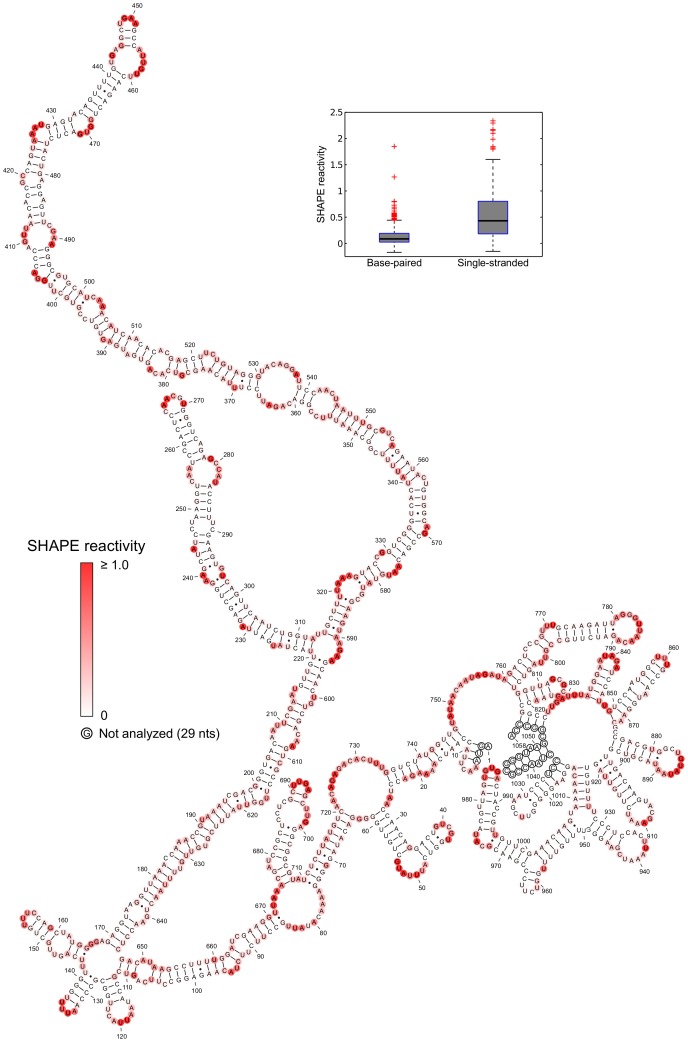
SHAPE-restrained secondary structure model for free STMV RNA. Nucleotides are colored according to their SHAPE reactivity (see scale). Inset shows a box plot comparing the distribution of SHAPE reactivity values between base paired and single-stranded nucleotides. Each grey box represents the interquartile range (IQR) of the data; the bottom and top edges of the box are the 25th and 75th percentiles, respectively. The band near the middle of each box is the median value. The whiskers above and below each box extend to the most extreme data points not considered outliers. Outliers are plotted individually as crosses. Points are outliers if they are greater than 1.5 IQR from the 75th percentile or less than 1.5 IQR from the 25th percentile. In this secondary structure model, the distribution for base paired nucleotides is narrower and has a much lower median value than the distribution for single-stranded nucleotides.

The SHAPE reactivity information was incorporated into the thermodynamic folding algorithm RNAstructure [18] as a pseudo-free energy change term [13] to predict a secondary structure model for the free STMV RNA (Figure 2). In the virus, it has been proposed that there are 30 stem-loops [4]. This proposal was incorporated into the Schroeder model by prohibiting long-range base pairing [7]. We imposed no restriction on the distance along the primary sequence between base-paired nucleotides, since there is no *a priori* reason for doing so for an RNA probed *in vitro*.

We recognize that chemical probing cannot define a single secondary structure [19], [20], because SHAPE reactivity is inversely correlated with base pairing, but the correlation is not perfect; some base paired nucleotides are reactive, and some unpaired nucleotides are not. To address this issue, we report the structure that is most consistent with the SHAPE data (Figure 2), along with several suboptimal structures (Figure S4), also generated by RNAstructure.

We evaluated the agreement between the model and the data by comparing the distribution of reactivity values in single-stranded nucleotides with the distribution of reactivities in base paired nucleotides (Figure 2, inset box plot). The reactivities of base paired nucleotides are less disperse and have a much lower median value than the reactivities of single-stranded nucleotides. These distributions are consistent with SHAPE experiments on RNAs with known secondary structures [21].

**Figure 3:**
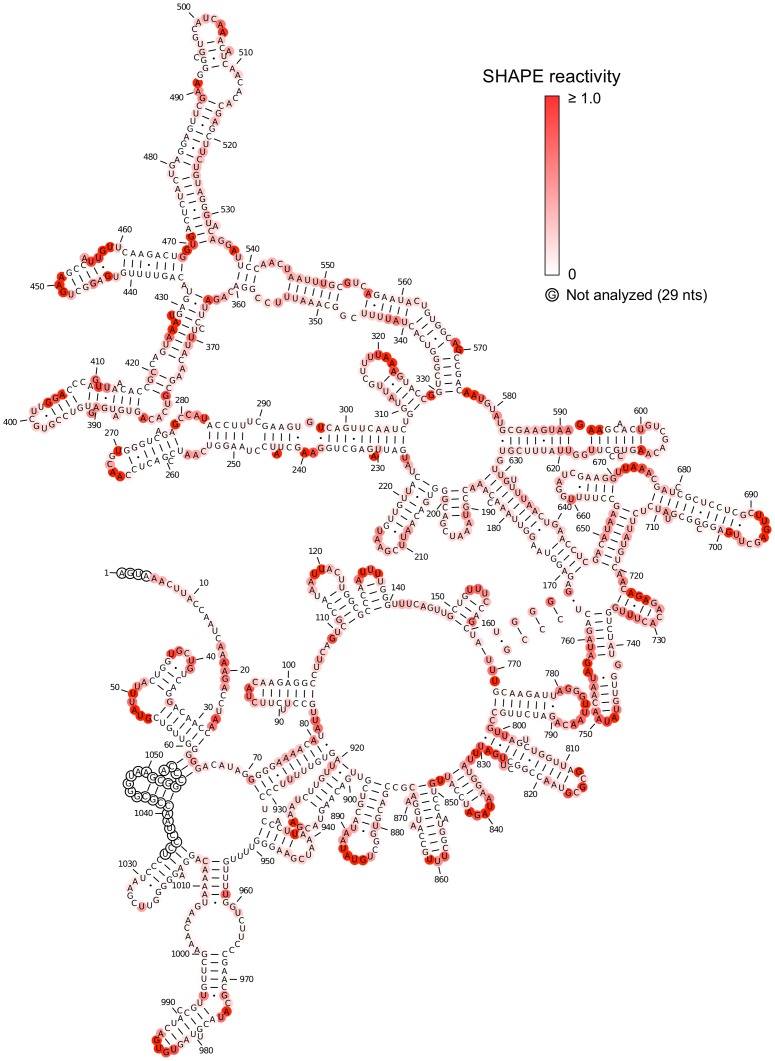
Minimum free energy (MFE) structure obtained for STMV RNA without the SHAPE data. The structure was predicted using RNAstructure with default parameters. Nucleotides are colored according to their SHAPE reactivity (see scale). The SHAPE data are not consistent with this model, since several base paired regions have high reactivity values.

The SHAPE-restrained secondary structure is characterized by significant long-range base pairing and minimal branching, especially for the region between nucleotides 169 and 646. This region, consisting of double-helical segments broken intermittently by small internal loops and bulges, is reminiscent of *in vitro* transcribed viroid RNA [22]. The SHAPE-restrained structure is noticeably different from the minimum free energy (MFE) structure (Figure 3) predicted using RNAstructure [18]. Unsurprisingly, the MFE structure is less consistent with the SHAPE data.

### Maximum Ladder Distance of the SHAPE-restrained STMV RNA Secondary Structure is Much Larger than Expected

The SHAPE-restrained secondary structure of STMV RNA appears unusually highly extended. To evaluate the extendedness of this secondary structure, we used a metric first introduced by Yoffe *et al.* [23], the maximum ladder distance (MLD). MLD is the largest value of ladder distance, *LD_ij_*, for all combinations of *i* and *j*, where *LD_ij_* is the number of base pairs that are crossed along the most direct path from base *i* to base *j* in the standard two-dimensional graph representing the secondary structure. Yoffe *et al.* previously used this measure to compare RNAs of *T* = 3 icosahedral viruses with a set of random RNA sequences with virus-like compositions [23]. For a given RNA sequence, they generated an ensemble of secondary structures, calculated the MLD for each of these and reported the average, designated 〈*MLD*〉. As a control, they generated an ensemble of secondary structures from shuffled sequences and calculated the 〈*MLD*〉 for that ensemble. They found that the RNA genomes of self-assembling icosahedral viruses have smaller 〈*MLD*〉 values than do shuffled sequences, *i.e.*, viral RNA secondary structures are predicted to be more highly branched than those of random sequences. They suggested that these viral RNAs would therefore have compact three-dimensional structures, facilitating viral assembly.

**Figure 4:**
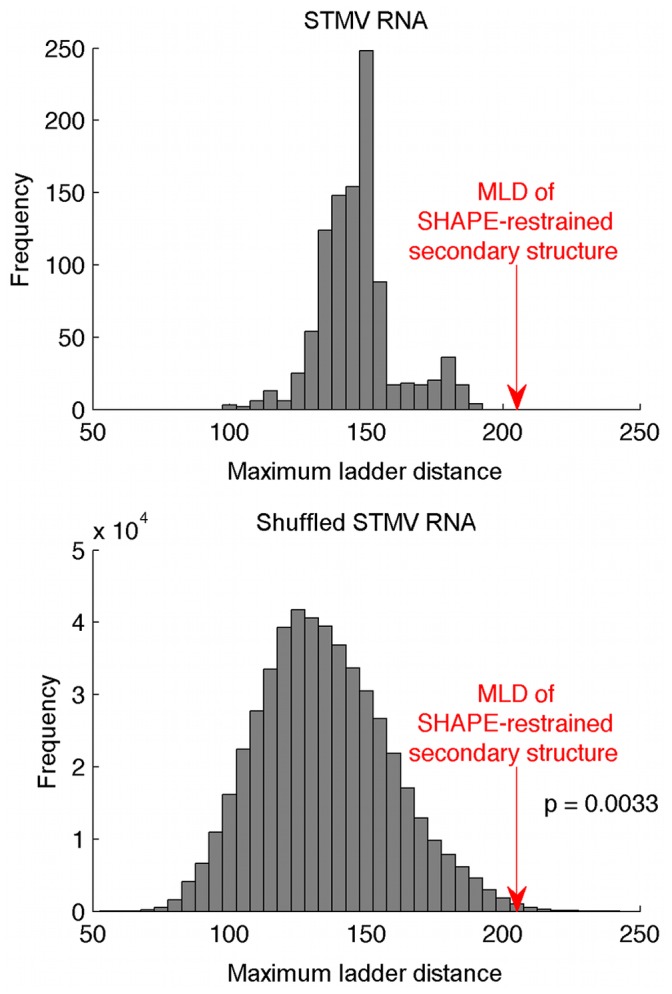
Histogram of maximum ladder distance values calculated for STMV RNA and shuffled STMV RNA sequences. The MLD of the SHAPE-restrained structure is much higher than the MLDs of 1000 suboptimal structures predicted for the STMV RNA sequence (top). The extreme MLD of the SHAPE-restrained structure is unlikely to have occurred by chance: the bottom histogram was obtained using 1000 suboptimal structures for each of 500 randomly shuffled sequences with the same length and nucleotide composition as STMV. Fewer than 0.4% of these structures have MLDs greater than the MLD of the SHAPE-restrained STMV structure.

The MLD of the SHAPE-restrained secondary structure (Figure 2) is 205. For comparison, the MLD of the more branched MFE structure (Figure 3) is 101, while 〈*MLD*〉 = 146.7 for a collection of 1000 suboptimal structures. Remarkably, the experimental MLD is higher than the MLD of any of the suboptimal structures (Figure 4, top panel). We estimated the probability distribution for MLD values of random RNAs with the same length and nucleotide composition as STMV (Figure 4, bottom panel), finding that it is highly unlikely that a secondary structure with an MLD this high would have occurred by chance (P<0.004).

We have also examined the MLDs of a series of suboptimal SHAPE-restrained structures, generated by RNAstructure (Figure S4). The first five of these all have similar, highly elongated structures, with MLDs of 169 or greater; the pseudoenergies of these structures range from −798 kcal/mol for the structure in Figure 2, to −784 kcal/mol for the fifth suboptimal structure. Structures with shorter MLDs (≤124) all have higher pseudoenergies (−770 kcal/mol or above), so they are clearly inconsistent with the SHAPE data.

This model of the STMV RNA secondary structure is at variance with the observation of Yoffe *et al*. that RNAs of small icosahedral viruses have smaller MLDs than do random sequences. We note, however, that their observations were based on data for *T* = 3 viruses with RNA genomes with lengths greater than 2500 nucleotides, while STMV is a *T* = 1 virus with a much smaller genome. Furthermore, it has been argued that STMV assembles as the RNA is replicated [5]. If so, then the 〈*MLD*〉 of STMV RNA is not relevant for assembly, since the RNA would not be in thermodynamic equilibrium, an implicit assumption made by Yoffe *et al*.

### SHAPE Probing Supports a tRNA-like Structure (TLS) at the 3′ End of STMV RNA

The 240 3′-terminal nucleotides of STMV RNA have more than 65% overall sequence similarity with the corresponding nucleotides of TMV U1 RNA, including two nearly identical regions of approximately 40–50 bases each [24]. On the basis of phylogenetic comparisons, Felden *et al.* proposed that the 3′ end of STMV RNA folds into a tRNA-like structure similar to that found in TMV RNA [9]. The authors also demonstrated that the STMV RNA could be aminoacylated *in vitro* with histidine, although STMV RNA charging is less efficient than TMV RNA.

**Figure 5:**
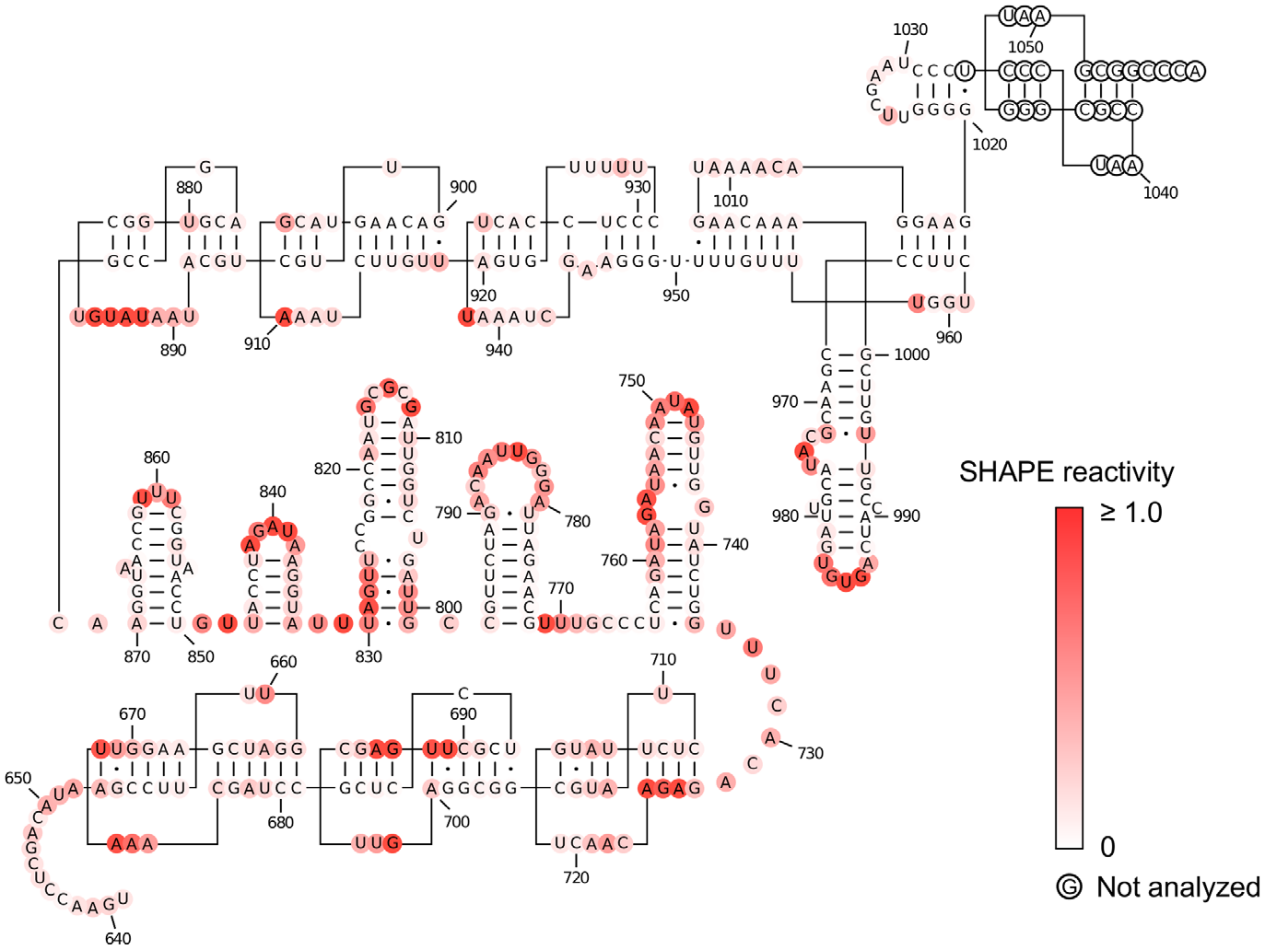
Predicted secondary structure at the 3′ end of STMV RNA. Secondary structure for the 406 3′-terminal nucleotides of STMV RNA proposed by Gultyaev *et al.* [10]. Nucleotides are colored according to their SHAPE reactivity (see scale). The SHAPE data supports the tRNA-like structure and the five stem-loops (nucleotides 728–1058), but does not support the second pseudoknot domain (nucleotides 653–727).

In a related study, Gultyaev *et al.* predicted a secondary structure for the 406 3′-terminal nucleotides of STMV RNA [10]. In addition to a tRNA-like structure at nucleotides 873–1058, their model included a stretch of three consecutive pseudoknots at nucleotides 653–727 and five stem-loops at nucleotides 735–870. Our SHAPE data support the predicted tRNA-like structure and the five stem-loops, but they are mostly inconsistent with the predicted pseudoknots at nucleotides 653–727 (Figure 5). It is important to note that the last 25 nucleotides at the 3′ end are missing in our analysis due to experimental limitations.

**Figure 6:**
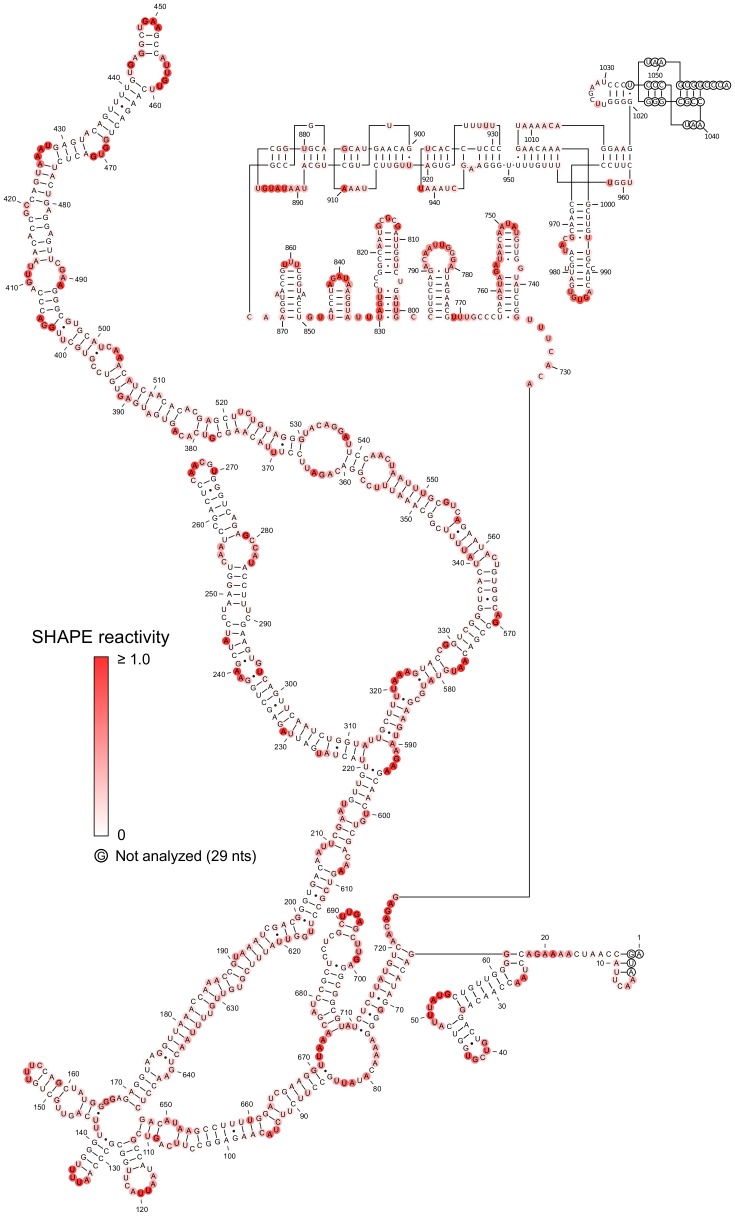
SHAPE-restrained secondary structure of free STMV RNA with a tRNA-like fold at the 3′ end. This alternate model of the STMV RNA was obtained by combining the SHAPE-restrained secondary structure predicted separately for nucleotides 1–727 (Figure 2) with the Gultyaev *et al.* prediction [10] for nucleotides 728–1058 (Figure 5). Nucleotides are colored according to their SHAPE reactivity (see scale). The extended central domain (nucleotides 64–720) is identical to that of Figure 2.

Since the RNAstructure program does not allow pseudoknots in its calculations, the tRNA-like structure and associated pseudoknots would not show up in any SHAPE-restrained secondary structure prediction of STMV RNA. Therefore, we built an alternate model of the genome by combining the SHAPE-restrained secondary structure predicted separately for nucleotides 1–727 with the Gultyaev prediction for nucleotides 728–1058 (Figure 6). This produces structures for the 5′ and 3′ ends of the RNA that differ from the structure shown in Figure 2, but the very extended central domain (nucleotides 64–720) is identical to that of Figure 2. We favor the model that includes the TLS (Figure 6) over the structure in Figure 2, because of the biochemical data [9].

### Comparison of Probing Data on Free RNA with Data on Encapsidated RNA

We compared our SHAPE reactivity data obtained on *in vitro* transcribed RNA with the Schroeder *et al.* chemical probing data obtained on encapsidated RNA [7]. They reported the top 161 nucleotides modified with dimethyl sulfate (DMS), carbodiimide (CMCT), or kethoxal. Of these strongly modified nucleotides, 86 were unreactive to the SHAPE reagent, 42 were moderately reactive, and 33 were highly reactive. Although this seems like a significant amount of disagreement, SHAPE probing does not always completely agree with traditional base-reactive reagents such as DMS [11], [20]. Schroeder *et al.* tried SHAPE probing of the STMV RNA *in virio*, finding that the signal:noise ratio was significantly lower with this reagent than with DMS, CMCT and kethoxal; they attributed this in part to the lack of a quenching step for SHAPE probing, arguing that the SHAPE reagents probably continue to react with the RNA during extraction of the RNA from the viral particle. (See Supporting Information in reference [7].).

**Figure 7:**
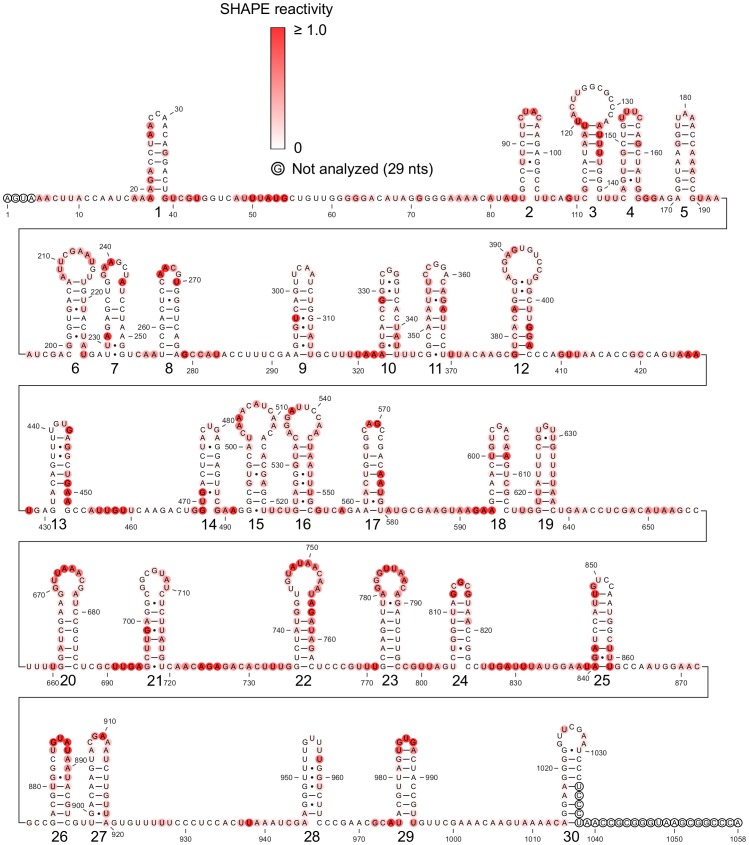
Schroeder secondary structure model for encapsidated STMV RNA. Schroeder *et al.* predicted this secondary structure on the basis of the co-replicational folding and assembly hypothesis, along with chemical probing data [7]. Nucleotides are colored according to their SHAPE reactivity (see scale), and the hairpin loops are numbered from 1 to 30. Hairpins 1, 3, 10–13, 17, 21–22, and 25 are clearly inconsistent with the SHAPE data.

Second, we compared our SHAPE data with the Schroeder model, finding that the agreement is not very good. In particular, Schroeder’s hairpins 1, 3, 10–13, 17, 21–22, and 25 are not consistent with the SHAPE data (Figure 7). This suggests that the secondary structure of the free RNA is different than the Schroeder model for the encapsidated RNA, as previously suggested [5]. Nor is this surprising: the Schroeder structure would not be stable in solution, as it has a very high folding free energy (−83 kcal/mol) relative to either the thermodynamic minimum free energy structure in Figure 3 (−331 kcal/mol) or the SHAPE-optimized structure in Figure 2 (−309 kcal/mol). When the RNA is packaged into the virus, if it must refold to this higher energy state, the cost would presumably be paid by favorable RNA-protein interactions.

**Figure 8:**
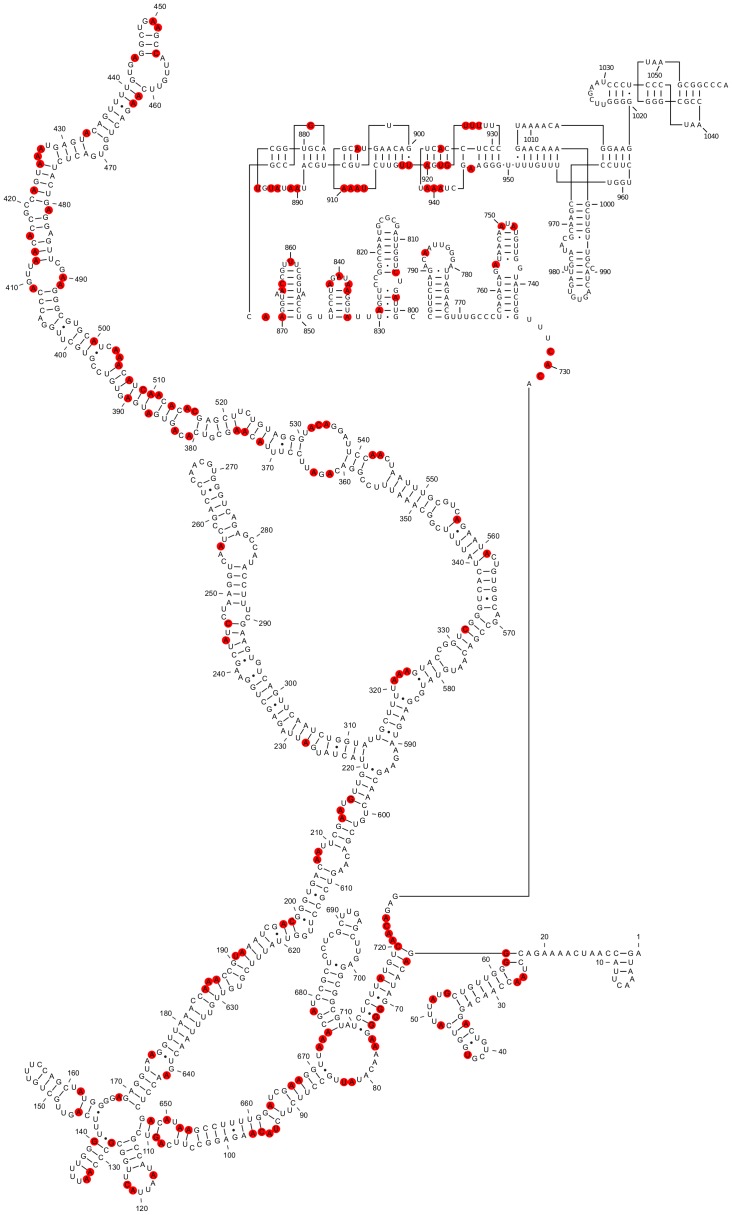
Mapping the chemical probing data from Schroeder *et al.* [7] onto the SHAPE-restrained secondary structure of *in vitro* transcribed STMV RNA. Red circles indicate nucleotides modified by DMS, kethoxal, or CMCT. The data do not appear to clearly rule out the proposed secondary structure of residues 1–730. A substantial number of the modifications occur in predicted loops, bulges, and single-stranded regions (67 out of 119 hits). Many of the reactive base-paired nucleotides are in A-U or G-U base pairs immediately adjacent to a predicted bulge loop (*e.g.*, 128, 185, 187, 192, 213, 413–414, 556, 561, 652–653, 663, 675), while others (382–390 and 503–515) are in a predicted stem that has two bulges and has no run of more than three consecutive base pairs, so it should be prone to fraying.

As a separate comparison, we asked whether or not the probing data of Schroeder *et al.* are consistent with the SHAPE-restrained model. (We are curious about the possibility that the encapsidated structure might resemble our model.) It is not possible to make a rigorous comparison, because Schroeder’s data were obtained on the RNA in the mature virus, while our model represents the RNA free in solution. It is hard to evaluate how much the capsid might protect the RNA, and impossible to know which residues might be affected. It is also unclear to what extent encapsidation of a structure like ours might cause local structural disruptions. There appears to be a not unreasonable agreement between the Schroeder data and our model in the extended region (residues 1–730), and in the tRNA-like domain (Figure 8). In the extended region, the biggest disagreements lie in the stem composed of residues 384–394 and 505–514, although this is a weak stem, containing three shorter stems of only three base pairs each, separated by bulges. Otherwise, many of the hits lie in proposed bulges, or in A-U base pairs immediately adjacent to bulges. We are unable to reach a firm conclusion about what, if anything, the Schroeder data say about the possibility that this structure – or parts of it – are found in the mature virus.

### SHAPE Reactivity Data for Free STMV RNA with and without Mg^2+^ are not Significantly Different

**Figure 9:**
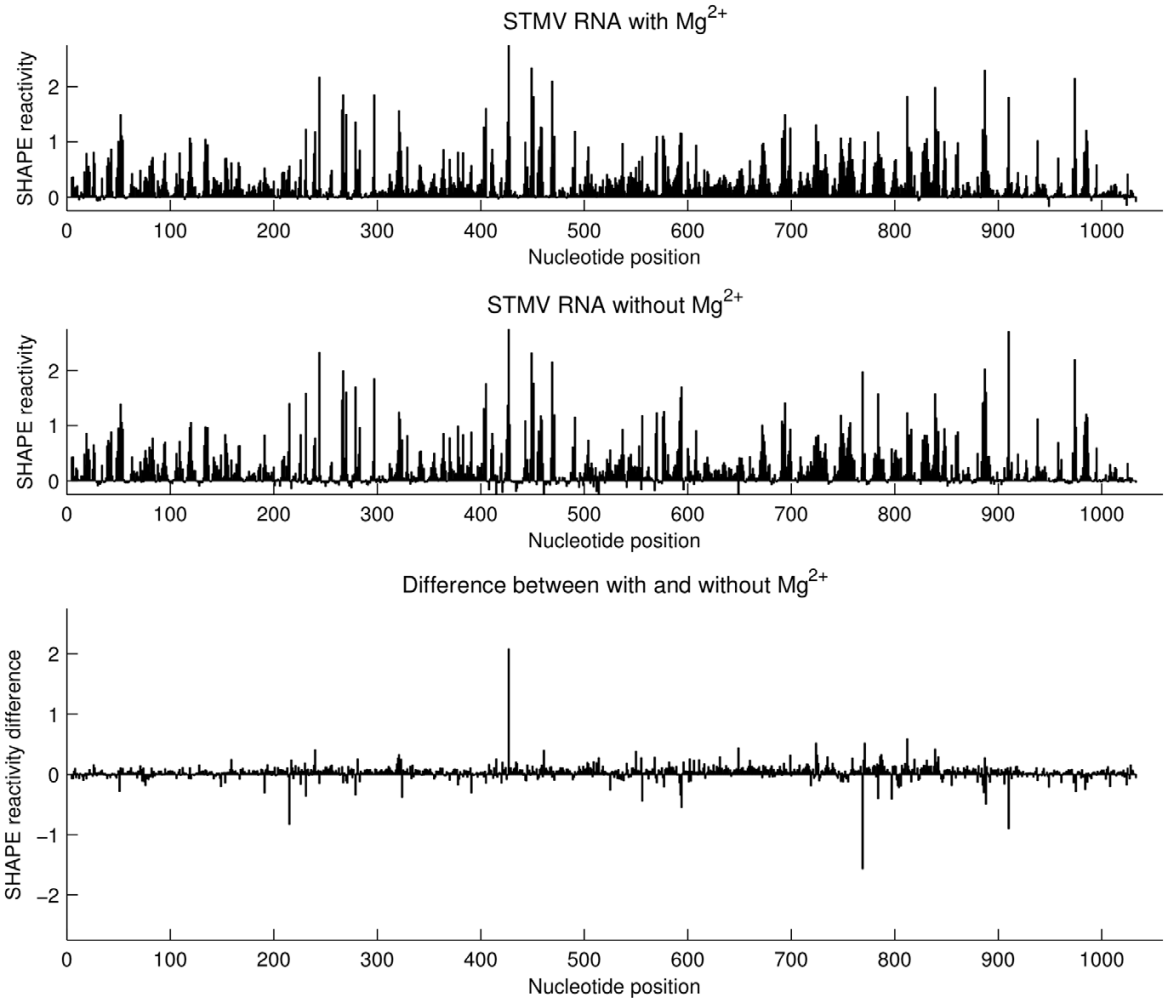
Effect of Mg^2+^ on the SHAPE reactivity profile of free STMV RNA. SHAPE reactivities for STMV RNA in the presence (top) and absence (middle) of Mg^2+^. The difference plot (bottom) shows that 10 mM Mg^2+^ has little effect on the SHAPE reactivity profile.

To examine the effect of Mg^2+^ on the folding of STMV RNA, we performed an otherwise identical SHAPE experiment on the RNA in the presence of 10 mM Mg^2+^. The presence of Mg^2+^ did not significantly change the SHAPE reactivity profile (Figure 9), indicating that STMV RNA folding is not dependent on Mg^2+^. Some RNAs, *e.g.*, tRNA, RNase P, the *Tetrahymena thermophila* group I intron P4–P6 domain, and domain III of the *T. thermophilus* 23S rRNA, show significant Mg^2+^-dependence of SHAPE reactivities [25]–[29]. STMV RNA is essentially an mRNA, so its folding is not necessarily expected to be dependent on Mg^2+^. Atomic force microscopy (AFM) images showed that STMV RNA that has been phenol-extracted from the intact virus exists in two temperature-dependent and reversible conformations, an open and a closed conformation [6]. Those authors suggested that secondary structure and significant tertiary interactions are maintained even at elevated temperature (65°C). Our SHAPE probing at 37°C suggests that either there are no significant tertiary interactions or, if there are, Mg^2+^ is not required for their formation.

### Biological Significance

The secondary structure proposed here (Figure 6) raises four questions.

First, is the structure of the *in vitro* transcribed RNA biologically relevant? A study by Mirkov *et al.* suggests that it is. They demonstrated that STMV RNA transcribed *in vitro* was biologically active, showing a consistent ability to infect tobacco plants also infected by TMV [30]. It is worth mentioning that STMV RNA can move systemically through a plant in both encapsidated and non-encapsidated forms [1], [31].

Second, does this structure play a role in viral assembly? It appears likely that the TLS represents a recognition signal for replication [9]. Also, the TLS at the 3′ end of brome mosaic virus (BMV) RNA has been shown to mediate icosahedral viral assembly and function as a simple telomere [32]–[35]. The STMV TLS might do the same.

Third, if this secondary structure is not that of the packaged RNA in the mature STMV virion, then what is its function? One plausible explanation is that it protects the RNA from degradation. Felden *et al.* have proposed that the tRNA-like structure (TLS) in STMV is essential for stability of its RNA [9], as has been demonstrated for TMV [36]. In addition, viroid RNAs (which are not encapsidated) have extended secondary structures, not unlike the extended domain in Figure 6. Wang *et al.* showed that “viroid and satellite RNAs are significantly resistant to RNA silencing-mediated degradation, suggesting that RNA silencing is an important selection pressure shaping the evolution of the secondary structures of these pathogens” [37]. This might well be the case for the extended domain of STMV RNA.

**Figure 10:**
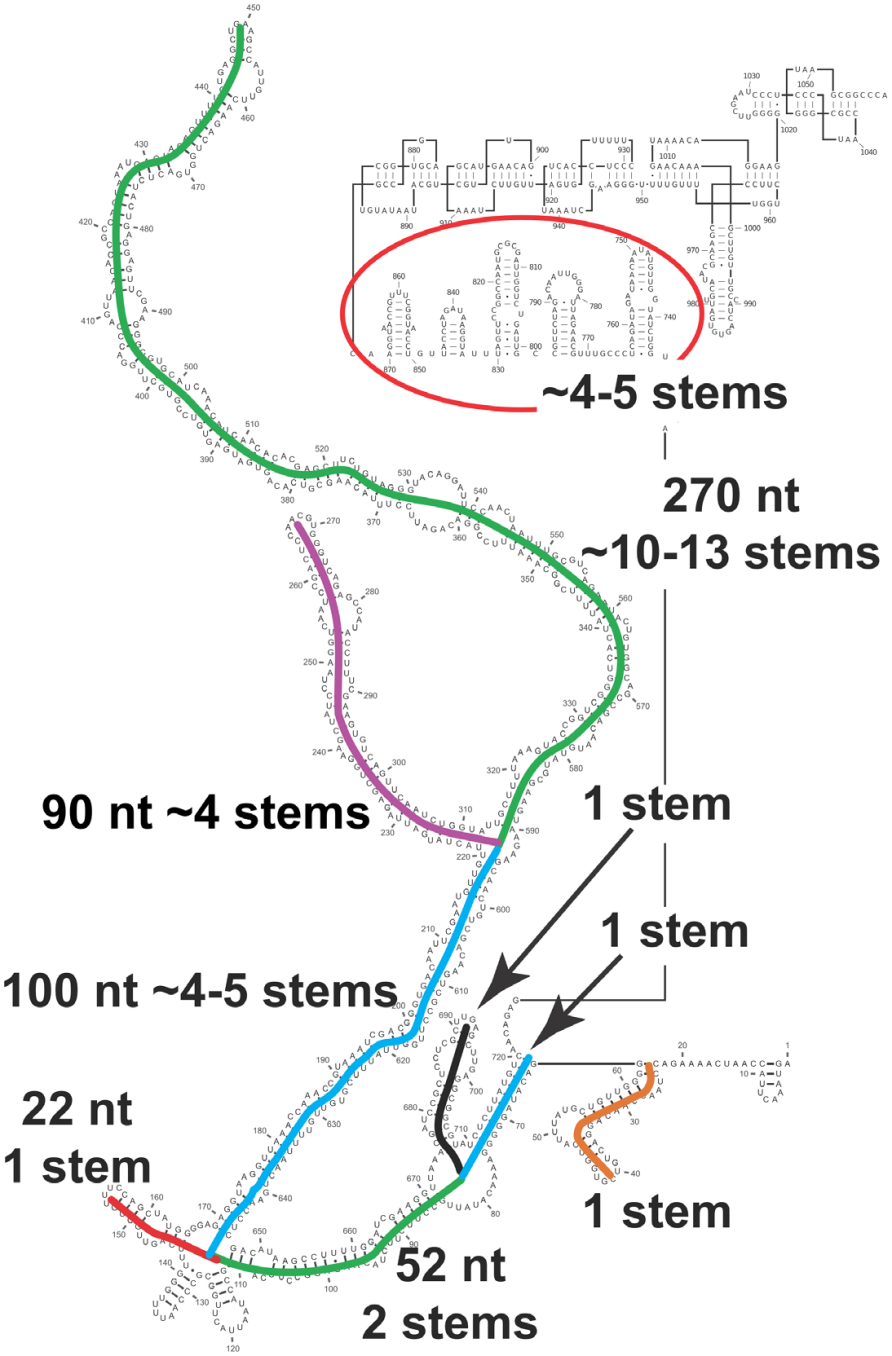
Identification of possible double-helical stems corresponding to those seen in the crystal structure. There are 30 stems in the crystal structure, each containing nine base pairs with an additional base stacked at each 3′ end, *i.e.*, 20 nucleotides (Figure 1). A model that connects successive stems would require something on the order of 5–10 nucleotides per connection. This figure shows how our secondary structure model might be organized to fit into the STMV capsid, with a sufficient number of stems to cover the 30 edges of the icosahedral frame, as required by Figure 1.

Finally, is it possible that this secondary structure is maintained inside the intact virion? As argued above, the chemical probing data from Schroeder *et al.* don’t give a firm answer to this question. Could the extended domain be arranged to cover the edges of the icosahedron, perhaps surrounding the tRNA-like structure in the core? Figure 10 shows how our model might be organized to provide a sufficient number of double-helical stems to do this.

### Conclusions

The SHAPE-restrained secondary structure of *in vitro* transcribed STMV RNA is highly extended, and the data support the predicted tRNA-like fold at the 3′ end of the RNA [9], [10]. Both of these features may stabilize the non-encapsidated RNA *in vivo*. The predicted secondary structure of the RNA transcribed *in vitro* is considerably different from that proposed for the genome in the intact virion [7]; we have previously developed an all-atom model of the mature virus based on the latter secondary structure [8]. Here we have suggested that it might also be possible to develop a model of the mature virus using the RNA secondary structure revealed by SHAPE probing, which corresponds to the equilibrium structure.

If the genomic RNA is packaged co-replicationally, as originally proposed [5], then the Schroeder secondary structure model [7] is probably correct. Alternatively, the RNA might be fully synthesized before packaging, achieving the structure that we have proposed (Figure 6). If this is the case, then either the RNA is packaged with our structure, or it undergoes extensive refolding to achieve the Schroeder structure. Additional experimental work is needed to determine the relationship between replication and packaging, and to identify the final structure of the viral genome after packaging into STMV.

## Methods

### Preparation of STMV RNA

STMV DNA appended with a 5′ T7 promoter and 3′ HindIII recognition sequence was synthesized by MWG Operon and provided in a pCR 2.1-TOPO plasmid. The plasmid was cleaved with PstI (New England Biolabs), gel purified, and religated to remove an extraneous T7 promoter. The plasmid was amplified in dH5α *Escherichia coli*, purified using the Endo-Free Plasmid Maxi kit (Qiagen), and sequenced bi-directionally (MWG Operon). This *in vitro* transcript runs as a single band in native gel electrophoresis (Figure S5), suggesting a single dominant conformation.

Transcription reactions were performed by the run-off method [38], using the MEGAscript High Yield Transcription Kit (Applied Biosystems). Plasmid containing the STMV gene was linearized with HindIII (New England Biolabs) and purified by DNA Clean & Concentrator Kit (Zymo Research). Linearized plasmid (∼0.5 µg) was transcribed in 20 µL reaction volumes for 2.5 hours at 37°C. RNA products from transcription reactions were recovered by ammonium acetate precipitation and resuspended in nuclease-free water (IDT). Yields were quantified by UV absorbance and purity by denaturing PAGE.

### SHAPE Probing of STMV RNA

SHAPE probing of STMV RNA was performed as described in [29]. Five 20-nt long DNA primers were used to primer reverse transcription reactions. The primers were labeled with 6-FAM at the 5′ end (Eurofins MWG Operon). The primers were named according to the most 5′ nucleotide of STMV RNA to which they anneal: 201, 5′-ACAACATTCGAATTGTCACC-3′; 411, 5′-TCATTTACTGGCGGTGTTAA-3′; 668, 5′-AGGAGCGGATCGTTTAACCT-3′; 831, 5′-ACAATGGATCTATTCCATAA-3′ and 1039, 5′-TGGGCCGCTTACCCGCGGTT-3′.

### SHAPE Data Processing

We converted the capillary electrophoresis (CE) data traces, or electropherograms, into SHAPE reactivities using in-house Matlab code. This procedure has been described in detail in Athavale *et al.* [29]. Briefly, this involved (1) aligning the traces to one another, (2) calculating and subtracting the baseline, (3) locating the peaks, (4) quantifying the area of each peak, (5) correcting for signal decay, (6) subtracting the background, and (7) normalizing. We used a new technique to correct for signal decay (see Methods S1, Table S1, Figure S1, Figure S2, and Figure S3 for details).

For the SHAPE data acquired on the RNA in 250 mM Na+ (no Mg^++^), the final reactivity values represent the average of nine separate datasets: three at a concentration of 3.25 mM NMIA, three at 6.5 mM NMIA, and three at 13 mM NMIA. For the SHAPE data acquired in 250 mM Na+ and 10 mM Mg2+, the final reactivity values represent the average of three separate datasets: one at a concentration of 3.25 mM NMIA, one at 6.5 mM NMIA, and one at 13 mM NMIA. As reported earlier [26], we have validated our methods by doing SHAPE experiments on the P4–P6 domain from the *Tetrahymena* Group I ribozyme, getting results that are similar to previous reports on the same molecule [25].

### RNA Secondary Structure Prediction

We folded the entire STMV RNA sequence (1058 nucleotides) using the thermodynamics-based free energy minimization algorithm in the RNAstructure software package, version 5.3 [18]. For the minimum free energy (MFE) structure, we used the default parameters. When calculating the SHAPE-restrained structure, we used the ‘-sh’ option to incorporate the SHAPE reactivities into the algorithm as restraints [13], [39], with default values for the SHAPE slope (2.6 kcal/mol) and SHAPE intercept (−0.8 kcal/mol). (We note that, since SHAPE reactivity penalizes single-strandedness for reactive nucleotides but does not absolutely prohibit base pairing, the SHAPE penalty is properly a restraint, rather than a constraint.).

### Maximum Ladder Distance Calculations

We calculated the MLD values using a C program (provided by Aron Yoffe and co-workers, UCLA). To compute the ensemble-average maximum ladder distance (〈*MLD*〉), we first generated a random sample of 1000 suboptimal structures, drawn with probabilities equal to their Boltzmann weights, using RNAsubopt, a program in the Vienna RNA software package, version 2.0 [40]. We then calculated the 〈*MLD*〉 as 
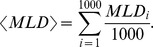


## Supporting Information

Figure S1 **Signal decay correction.** The regions of overlapping data from different primers are not on the same scale (top). After scaling all of the primers to one another such that the overlapping regions match up, the resulting signal decays rapidly (middle). After correcting for signal decay, the overlapping regions are in agreement (bottom).
(TIF)

Figure S2 **Quantitative correlation between peak area data in overlapping primer reads.** This demonstrates that signal decay in the regions of overlapping data is similar. Pearson’s *r*-values are shown.
(TIF)

Figure S3 **Combined peak area signal after decay correction.** The thick black line fitted to the corrected peak area data has a slope of zero, ensuring that intense values in the beginning, middle, and end of the signal are of uniform height.
(TIF)

Figure S4 **Predicted secondary structures for STMV RNA.** SHAPE MFE and Subopts #1–9 were predicted using the SHAPE experimental data as constraints. Default MFE was predicted without the SHAPE data. Each secondary structure is shown as an arc diagram, in which the sequence is arranged along a horizontal line and base pairs are shown as arcs connecting the corresponding bases. The structures are listed in order of ascending pseudo-energy values. Pseudo-energy is the calculated free energy that includes the SHAPE pseudo-energy terms. Also shown are the energy values evaluated using the default energy model parameters ignoring SHAPE terms. MLD is the maximum ladder distance. All structures predicted using *RNAstructure* version 5.3.
(TIF)

Figure S5 ***In vitro*** **transcribed STMV RNA runs as a single band on a native gel.** STMV RNA is run on a 1% agarose gel. No sample was loaded in lanes 2 or 4. Lanes 1 and 3 contain STMV RNA in SHAPE probing buffer without Mg^2+^ (50 mM HEPES pH 8.0, 200 mM sodium acetate pH 8.0) and lane 5 contains STMV RNA in 100 mM Tris-HCl pH 8.0. All samples were heated to 90°C for 2 min. Samples in lanes 1 and 5 were snap-cooled by chilling on ice, while the one in lane 3 was allowed to slow-cool to room temperature. The samples were then loaded on the gel using 6X native gel loading dye (New England Biolabs) and stained with SYBR Gold nucleic acid gel stain (Invitrogen). Lanes 1, 3 and 5 contain a single band, indicating a single dominant conformation.
(TIF)

Table S1 **Primers used to analyze the STMV RNA.**
(PDF)

Methods S1 (PDF)
