# Supplementary figures and images for: In Vitro Secondary Structure of the Genomic RNA of Satellite Tobacco Mosaic Virus
Source: PLoS One. 2013 Jan 22;8(1):e54384. doi: 10.1371/journal.pone.0054384 (PMC3551766; doi:10.1371/journal.pone.0054384)

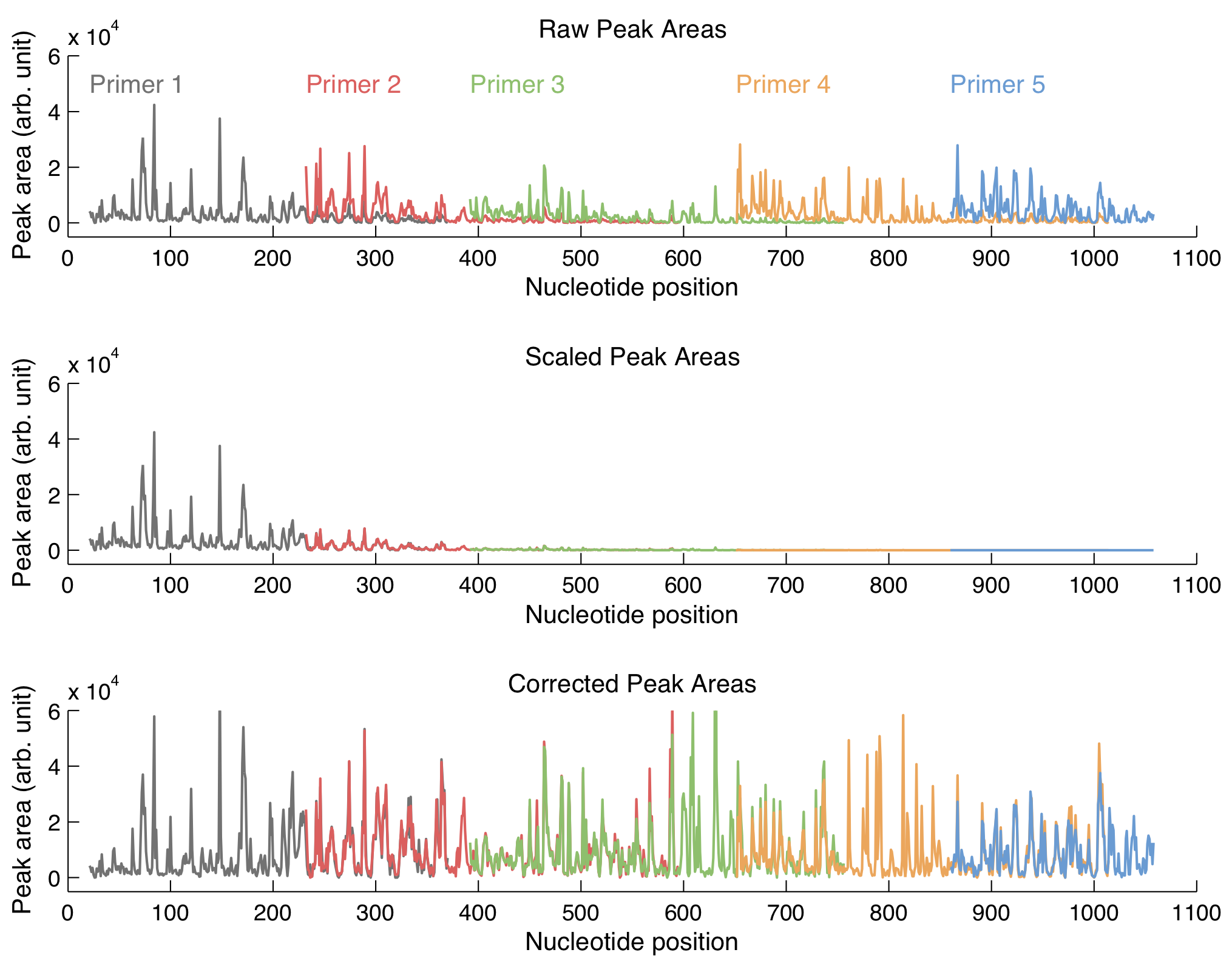

Supplement: Figure S1 — Signal decay correction. The regions of overlapping data from different primers are not on the same scale (top). After scaling all of the primers to one another such that the overlapping regions match up, the resulting signal decays rapidly (middle). After correcting for signal decay, the overlapping regions are in agreement (bottom). (TIF) [file pone.0054384.s001.tif]

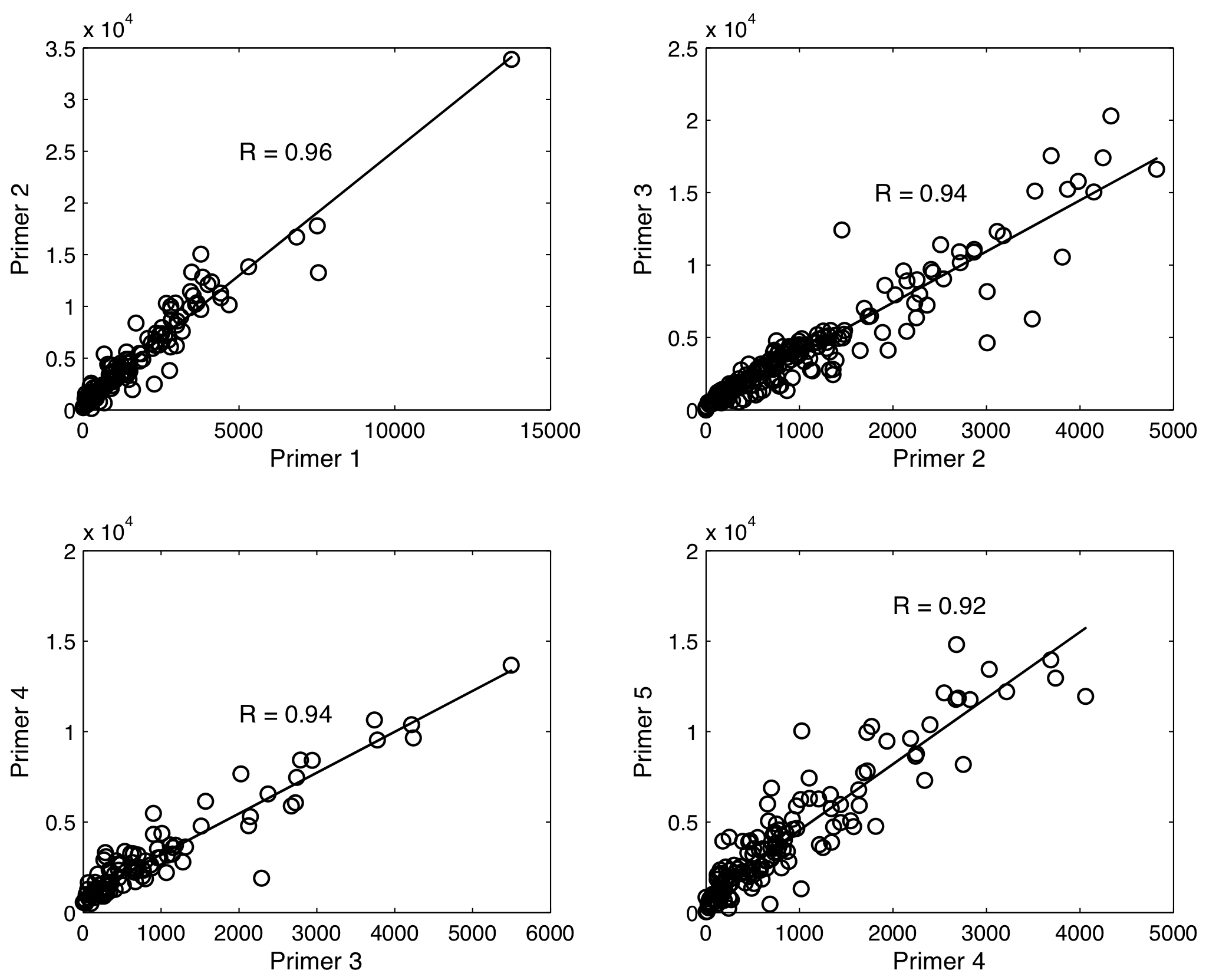

Supplement: Figure S2 — Quantitative correlation between peak area data in overlapping primer reads. This demonstrates that signal decay in the regions of overlapping data is similar. Pearson’s r-values are shown. (TIF) [file pone.0054384.s002.tif]

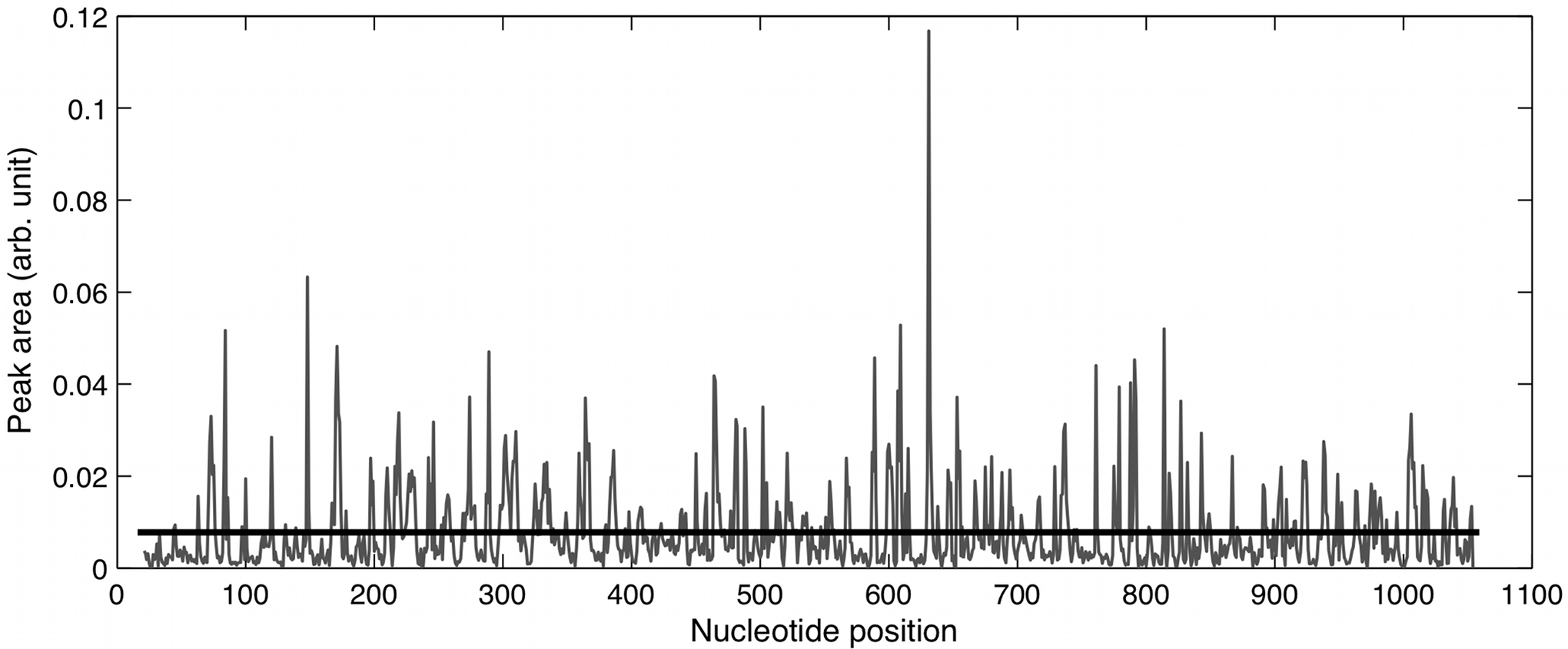

Supplement: Figure S3 — Combined peak area signal after decay correction. The thick black line fitted to the corrected peak area data has a slope of zero, ensuring that intense values in the beginning, middle, and end of the signal are of uniform height. (TIF) [file pone.0054384.s003.tif]

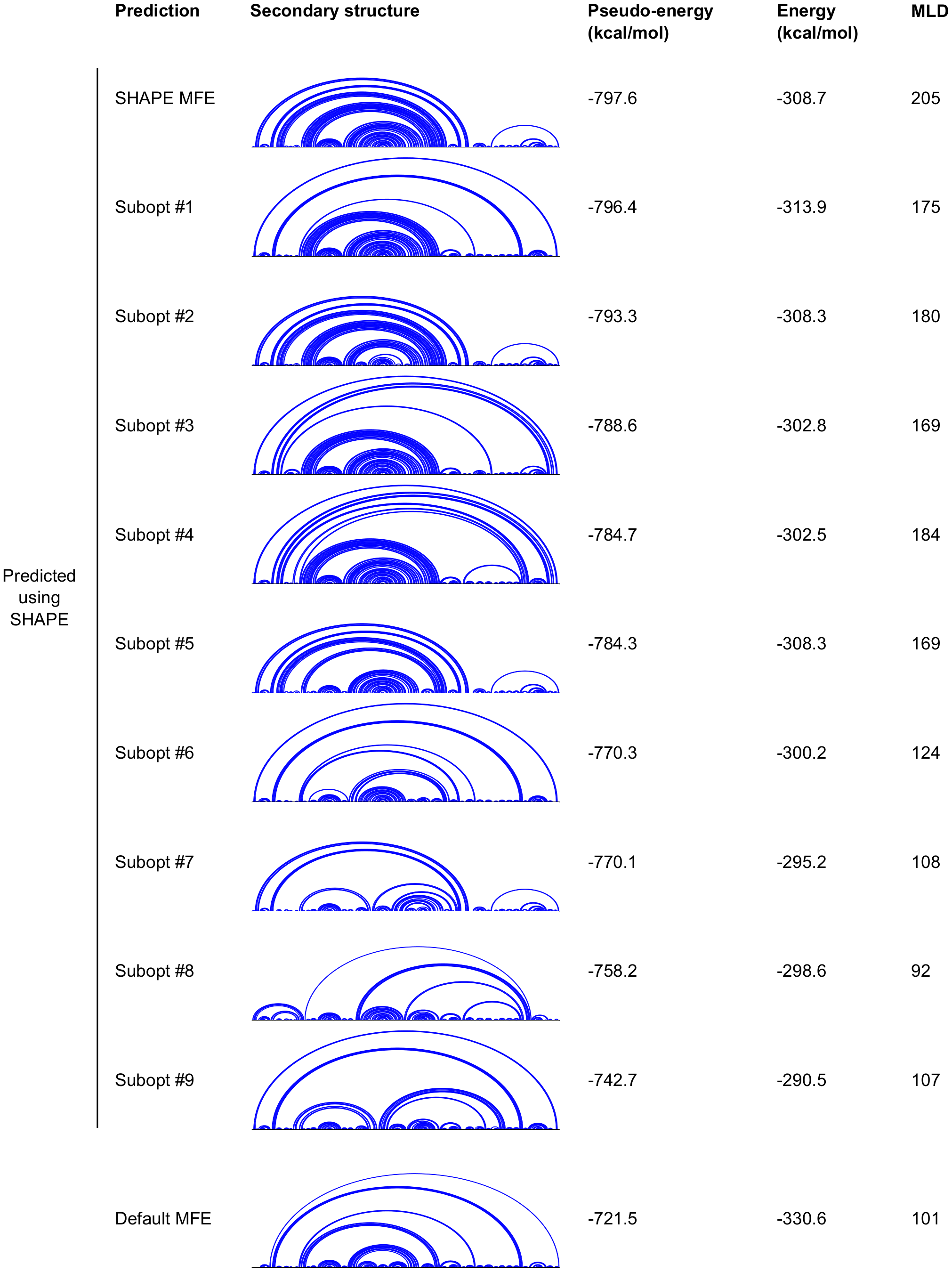

Supplement: Figure S4 — Predicted secondary structures for STMV RNA. SHAPE MFE and Subopts #1–9 were predicted using the SHAPE experimental data as constraints. Default MFE was predicted without the SHAPE data. Each secondary structure is shown as an arc diagram, in which the sequence is arranged along a horizontal line and base pairs are shown as arcs connecting the corresponding bases. The structures are listed in order of ascending pseudo-energy values. Pseudo-energy is the calculated free energy that includes the SHAPE pseudo-energy terms. Also shown are the energy values evaluated using the default energy model parameters ignoring SHAPE terms. MLD is the maximum ladder distance. All structures predicted using RNAstructure version 5.3. (TIF) [file pone.0054384.s004.tif]

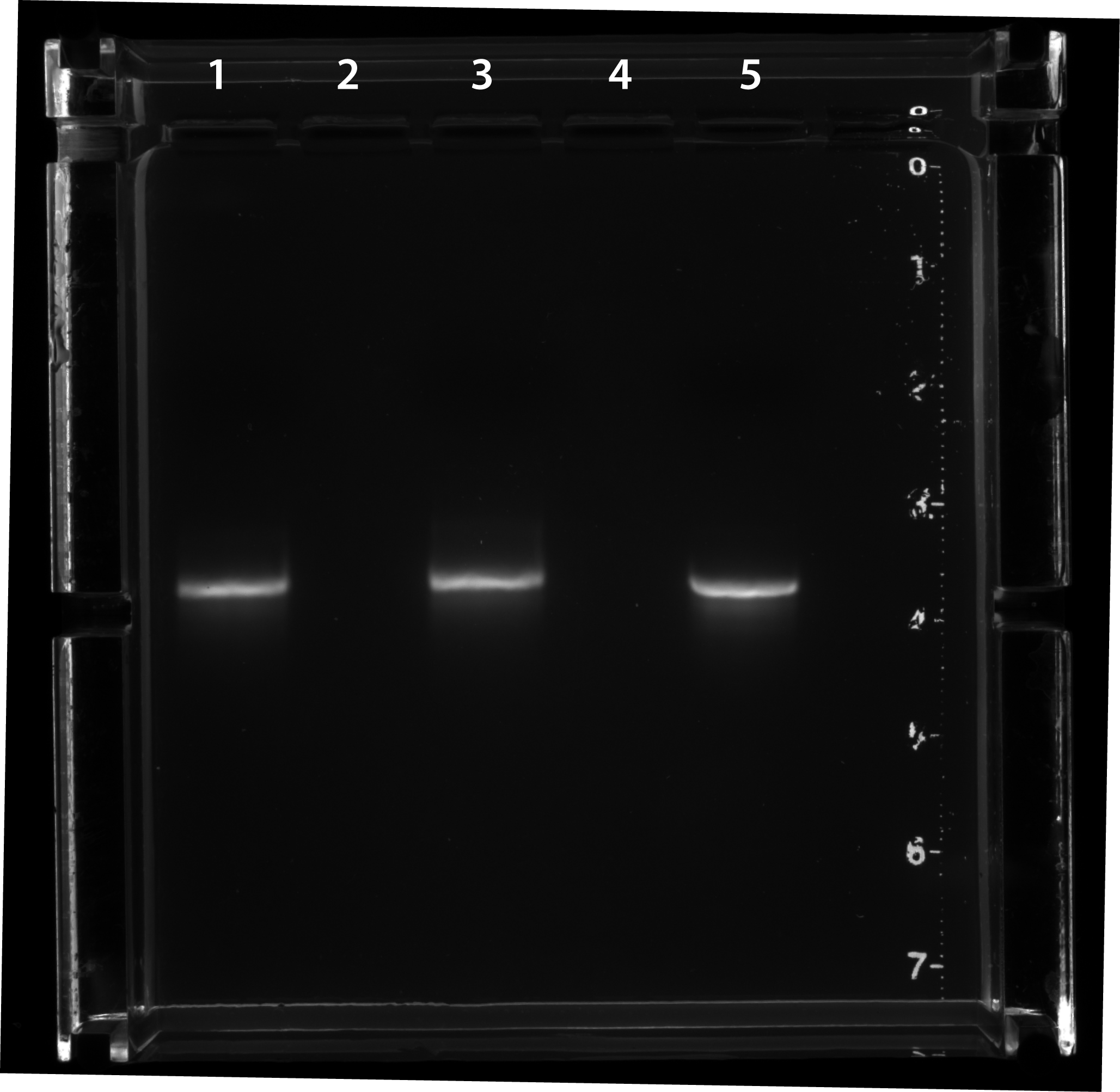

Supplement: Figure S5 — In vitro transcribed STMV RNA runs as a single band on a native gel. STMV RNA is run on a 1% agarose gel. No sample was loaded in lanes 2 or 4. Lanes 1 and 3 contain STMV RNA in SHAPE probing buffer without Mg2+ (50 mM HEPES pH 8.0, 200 mM sodium acetate pH 8.0) and lane 5 contains STMV RNA in 100 mM Tris-HCl pH 8.0. All samples were heated to 90°C for 2 min. Samples in lanes 1 and 5 were snap-cooled by chilling on ice, while the one in lane 3 was allowed to slow-cool to room temperature. The samples were then loaded on the gel using 6X native gel loading dye (New England Biolabs) and stained with SYBR Gold nucleic acid gel stain (Invitrogen). Lanes 1, 3 and 5 contain a single band, indicating a single dominant conformation. (TIF) [file pone.0054384.s005.tif]
