# Supplementary material for: In Vitro Secondary Structure of the Genomic RNA of Satellite Tobacco Mosaic Virus
Source: PLoS One. 2013 Jan 22;8(1):e54384. doi: 10.1371/journal.pone.0054384 (PMC3551766; doi:10.1371/journal.pone.0054384)
Supplement: Table S1 — Primers used to analyze the STMV RNA. (PDF) [file pone.0054384.s006.pdf]

Table S1

Primer	Primer annealing location	Nucleotides read (useable data)	Nucleotides used in combined signal
1	1-20	25-370	25-236
2	209-228	237-595	237-396
3	372-391	397-756	397-655
4	629-648	656-1014	656-864
5	839-858	865-1053	865-1053
