# Supplementary material for: In Vitro Secondary Structure of the Genomic RNA of Satellite Tobacco Mosaic Virus
Source: PLoS One. 2013 Jan 22;8(1):e54384. doi: 10.1371/journal.pone.0054384 (PMC3551766; doi:10.1371/journal.pone.0054384)
Supplement: Methods S1 — (PDF) [file pone.0054384.s007.pdf]

Methods S1

We used 5 DNA primers to analyze the STMV RNA. They were designed to anneal at roughly equally spaced positions along the sequence so that the combined primer extension reactions would span the entire RNA (Table S1). The primers were designed with the assistance of Primer3Plus [1]. Since we typically obtain read lengths of more than 300 nucleotides per primer extension reaction, the primer extension reactions for STMV RNA resulted in regions of overlapping data from different primers. These regions of overlapping data are important for our data processing procedure. Note that for this part of the procedure we number the nucleotides from 1 to 1058 with respect to the 3' end, not the 5' end.

When using multiple primers to analyze an RNA, the typical approach is to process the data from each primer extension reaction individually [2,3]. We take a similar approach here to convert the capillary electrophoresis data into raw peak areas. But after this step we deviate from the established protocol and combine the peak area data from all the primer extension reactions into one signal. We find it easier to complete the processing steps of correcting for signal decay, subtracting the background, and normalizing the data if we are working with one combined dataset.

We combine the data by taking advantage of the information contained in the regions of overlapping data. Plotting the peak area signals for all of the individual primer extension datasets on a single plot, we see that the data in the overlapping regions do not match up (Figure S1, top panel). In other words, the data from one primer extension reaction will be higher or lower than the one that it overlaps with. There are two reasons for this. First, the data from two different primer extension reactions will not in general be on the same scale due to experimental variations. Second, the signal for each primer extension reaction decays in an approximately exponential fashion for reasons that have been explained previously [4,5]. We observe here that whatever factors cause signal decay in one primer extension reaction should also apply to the other primer in the region of overlap. This is confirmed by computing Pearson's correlation coefficient for the peak areas in the

overlapping region between two different primer extension reads (Figure S2). As expected, we see a linear relationship. Therefore, we need only to apply a scaling factor to one of the primer datasets to have the overlapping regions match up. We do this by automatically finding the scaling factor that minimizes the sum of squares difference between the peak areas in one primer dataset and the corresponding peak areas in the overlapping primer dataset. For example, we scale the primer 2 data to the primer 1 data so that the peak areas match. Then we scale the primer 3 data to the scaled primer 2 data, and so on until we have scaled all the primer data (Figure S1, middle panel). To combine the data from all of the primers into one signal, we use data from each of the primers as shown in Table S1. We use primer 1 data up until the point primer 2 starts, and then we use primer 2 data up until the point primer 3 starts, and so on. There are other ways of combining the data, for example by taking the average of the values in the overlapping region. The combined dataset spans nucleotides 25 to 1053.

Next we perform signal decay correction on the combined dataset. Rather than fitting the data to an exponential function, we use a nonparametric correction factor developed by Aviran *et al.* for modeling polymerase drop-off [4]. The corrected peak area, Y_k, is calculated as follows:

$$Y_k = \log\left(1 - \frac{X_k}{\sum_{i=k}^{n+1} X_i}\right)$$

where n is the number of nucleotides and X_k is the raw peak area for nucleotide k for $k=1,\ldots,n$. The amount of full-length transcript is represented by X_{n+1}. Since we are not generally able to quantify the amount of full-length transcript, we must approximate its value. We do this by fitting a straight line to the corrected data and choosing the value for X_{n+1} that results in a line with a slope of zero (Figure S3). The intense values in the beginning, middle, and end of the signal are thus of uniform height [5].

The remaining data processing steps are performed as described previously [6].

References

1. Untergasser A, Nijveen H, Rao X, Bisseling T, Geurts R, et al. (2007) Primer3Plus, an enhanced web interface to Primer3. Nucleic Acids Res 35: W71-74.
2. Deigan KE, Li TW, Mathews DH, Weeks KM (2009) Accurate SHAPE-directed RNA structure determination. Proc Natl Acad Sci U S A 106: 97-102.
3. Watts JM, Dang KK, Gorelick RJ, Leonard CW, Bess JW, Jr., et al. (2009) Architecture and secondary structure of an entire HIV-1 RNA genome. Nature 460: 711-716.
4. Aviran S, Trapnell C, Lucks JB, Mortimer SA, Luo S, et al. (2011) Modeling and automation of sequencing-based characterization of RNA structure. Proceedings of the National Academy of Sciences of the United States of America 108: 11069-11074.
5. Vasa SM, Guex N, Wilkinson KA, Weeks KM, Giddings MC (2008) ShapeFinder: a software system for high-throughput quantitative analysis of nucleic acid reactivity information resolved by capillary electrophoresis. RNA 14: 1979-1990.
6. Athavale SS, Gossett JJ, Hsiao C, Bowman JC, O'Neill E, et al. (2012) Domain III of the T. thermophilus 23S rRNA folds independently to a near-native state. RNA 18: 752-758.
